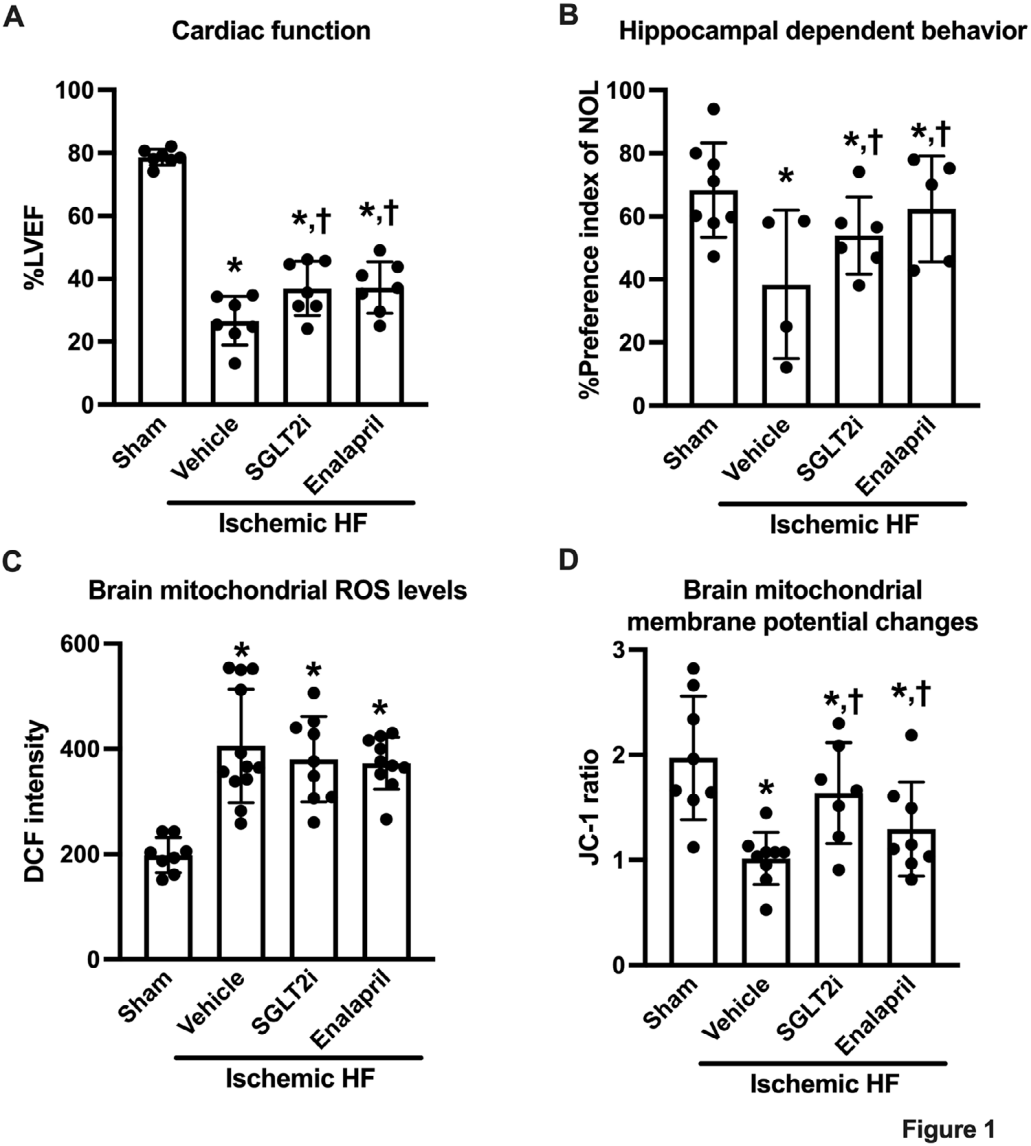# Sodium glucose transporter 2 inhibitor alleviates cognitive impairment in rats with ischemic heart failure

**DOI:** 10.1002/alz.084894

**Published:** 2025-01-03

**Authors:** Nattayaporn Apaijai, Tanawat Attachaipanich, Chayadom Maneechote, Busarin Arunsak, Apisek Kongkaew, Nipon Chattipakorn, Siriporn C Chattipakorn

**Affiliations:** ^1^ Neurophysiology Unit, Cardiac Electrophysiology Research and Training Center, Faculty of medicine, Chiang Mai University, Chiang Mai, Thailand, Chiang Mai Thailand; ^2^ Cardiac Electrophysiology Research and Training Center, Faculty of Medicine, Chiang Mai University, Muang, Chiang Mai Thailand; ^3^ Center of Excellence in Cardiac Electrophysiology Research, Chiang Mai University, Chiang Mai Thailand; ^4^ Neurophysiology Unit, Cardiac Electrophysiology Research and Training Center, Faculty of Medicine, Chiang Mai University, Chiang Mai Thailand; ^5^ Animal House Unit, Faculty of Medicine, Chiang Mai University, Muang, Chiang Mai Thailand

## Abstract

**Background:**

Sodium glucose transporter 2 inhibitor (SGLT2i) is the latest guideline‐directed medical therapy for patients with heart failure, as it has demonstrated favorable cardiovascular outcomes in heart failure (HF) patients with or without diabetes. Furthermore, SGLT2i has effectively improved cognitive function in older adults with diabetes and HF. However, the effects of SGLT2i on cognitive function and brain mitochondrial function in rats with ischemic HF have never been investigated.

**Method:**

Male rats underwent left anterior descending coronary artery ligation to induce myocardial ischemia or sham operation. Heart failure (HF) was confirmed after one week of surgery by a reduction of left ventricular ejection fraction less than 50%. Subsequently, HF rats were divided into three groups to receive: 1) Vehicle, 2) SGLT2i (dapagliflozin; 1 mg/kg), and 3) Enalapril (10 mg/kg) for 8 weeks. Then, cognitive function and brain mitochondrial function were then determined.

**Result:**

The ischemic HF rats exhibited a reduced LVEF compared to the sham group, and treatment with dapagliflozin and enalapril effectively improved LVEF compared to the vehicle group. Cognitive impairment was observed in the ischemic HF rats, and this was associated with brain mitochondrial dysfunction, as indicated by increased mitochondrial oxidative stress, mitochondrial membrane depolarization, and mitochondrial swelling. Treatment with dapagliflozin showed a similar degree of improved cognitive function as enalapril. We found that although both drugs did not reduce brain mitochondrial oxidative stress, they effectively reduced brain mitochondrial membrane depolarization and mitochondrial swelling in ischemic HF rats (p<0.05; **Figure 1**).

**Conclusion:**

SGLT2i and enalapril effectively improved cognitive function by reducing brain mitochondrial dysfunction in ischemic HF rats.